# Reduction of conspicuous facial pores by topical fullerene: possible role in the suppression of PGE_2_ production in the skin

**DOI:** 10.1186/1477-3155-12-6

**Published:** 2014-02-22

**Authors:** Shigeki Inui, Ayako Mori, Masayuki Ito, Sayuri Hyodo, Satoshi Itami

**Affiliations:** 1 Department of Regenerative Dermatology, Graduate School of Medicine, Osaka University, 2-2, G2, Yamadaoka, Suita -shi, Osaka 565-0871, Japan; 2 Clinic Mori, 1-1-5, Motoakasaka, Minato-ku, Tokyo 107-0051, Japan; 3 Vitamin C60 BioResearch Corporation, 1-3-19, Yaesu, Chuo-ku, Tokyo 103-0028, Japan

**Keywords:** Fullerene, Facial pore, Prostaglandin E_2_, Cosmeceutical, Melanin

## Abstract

**Background:**

Conspicuous facial pores are therapeutic targets for cosmeceuticals. Here we examine the effect of topical fullerene on conspicuous facial pores using a new image analyser called the VISIA^®^ system. Ten healthy Japanese females participated in this study, and they received applications of 1% fullerene lotion to the face twice a day for 8 weeks.

**Findings:**

Fullerene lotion significantly decreased conspicuous pores by 17.6% (p < 0.05, Wilcoxon signed-rank test) after an 8-week treatment. A self-administered questionnaire indicated that this reduction achieved cosmetically appreciable effects. In addition, to investigate the mechanism of effect of fullerene, we examined its effect on UVB-induced prostaglandin E_2_ (PGE_2_) production in reconstructed human epidermis (RhE). The results showed that irradiation of RhE with 1000 mJ/cm^2^ increased PGE_2_ production by 62.3% (p < 0.05, Mann-Whitney *U*-test) and the addition of 28 μM fullerene significantly suppressed the UVB-induced PGE_2_ production by 18.3% (p < 0.05).

**Conclusions:**

Fullerene lotion significantly decreases conspicuous facial pores after an 8-week treatment possibly through the suppression of PGE_2_ production in the epidermis.

## Background

Conspicuous facial pores are therapeutic targets for cosmeceuticals. Previously it has been reported that the topical vitamin C derivative ascorbyl 2-phosphate 6-palmitate (APPS) decreased conspicuous facial pores [1]. Here we examine the effect of topical fullerene on conspicuous facial pores using the new image analszer called the VISIA^®^ system (Canfield Scientific Inc., Fairfield, NJ).

## Methods

Ten healthy Japanese females participated in this study between January and June of 2013 at Clinic Mori (Minato-ku, Tokyo). Age range of participants was from 39 to 70 years and the mean age was 46 years. After an informed consent was obtained, 1% fullerene lotion (Radical Sponge^®^ 1.0%, 2-phenoxyethanol 0.5%, glycerine 5.0%, 1,3-butylene glycol 5.0%, essential oil as needed, water q.s. to 100%) was applied on the face twice a day for 8 weeks. The subjects were not permitted to use sunscreen products. However, they were allowed to use ordinary cosmetics but not medicinal cosmetics. The high-resolution analysing system VISIA-CR (Canfield, Fairfield, NJ) was used for computer analysis to precisely measure conspicuous pores through analysis scripts [2]. The photographs from VISIA-CR were analysed by a pores analysis algorithm in Vaestro image analysis toolkit, v 2.0 (Canfield, Fairfield, NJ) and subsequently the visible conspicuous facial pores were quantitatively measured. The analysis was carried out separately on the right and left sides of the face and the results were combined to get the total pore count. In principle, the pores were recognised as circular areas darker than the surrounding skin tone and much smaller than skin spots. This study was approved by the institutional ethics committee. An informed consent was obtained from all patients before enrolment in the study. To examine the effect of fullerene on UVB-induced prostaglandin E_2_ (PGE_2_) production in reconstructed human epidermis (RhE) LabCyte EPI-MODEL24 (Japan Tissue Engineering Co. Ltd, Gamagori, Japan), RhE was incubated in 500 μl of the assay medium of the kit on the 24-well plate for 1 hour at 37°C in a humidified atmosphere containing 5% CO_2._ Then, 2.8 or 28 μM water-soluble polyvinylpyrrolidone (PVP)-wrapped fullerene or a mock solution (1% PVP in PBS) was added. After incubation for 1 hour, the cultures were irradiated with 1000 mJ/cm^2^ UVB. Thirty minutes later, the medium was aspirated and the cells were washed three times with PBS. The cultures were kept in the fresh medium for 6 hours and the conditioned medium was finally subjected to PGE_2_ ELISA (PGE_2_ EIA Monoclonal Kit, Cayman Chemical Company, Michigan). A statistical analysis was performed using Wilcoxon signed-rank test or Mann-Whitney *U*-test. A difference with a p value of <0.05 was considered significant.

## Results and discussion

**Figure 1:**
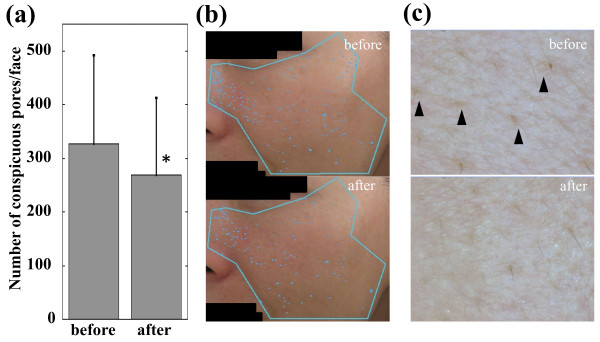
**Effect of fullerene on conspicuous facial pores. (a)** Reduction of the conspicuous facial pores after fullerene treatment for 8 weeks. The graph shows the mean ± standard deviation (n = 10). *p < 0.05, Wilcoxon signed-rank test. **(b)** The photos of conspicuous pores on the cheek by VISIA^®^ in the representative case (43-year-old female) before (upper panel) and after fullerene treatment for 8 weeks (lower panel) are shown. The number of conspicuous pores has decreased from 170 to 103. **(c)** Using dermoscopy, a regression of conspicuous pores (triangles) is observed in the same case.

**Figure 2:**
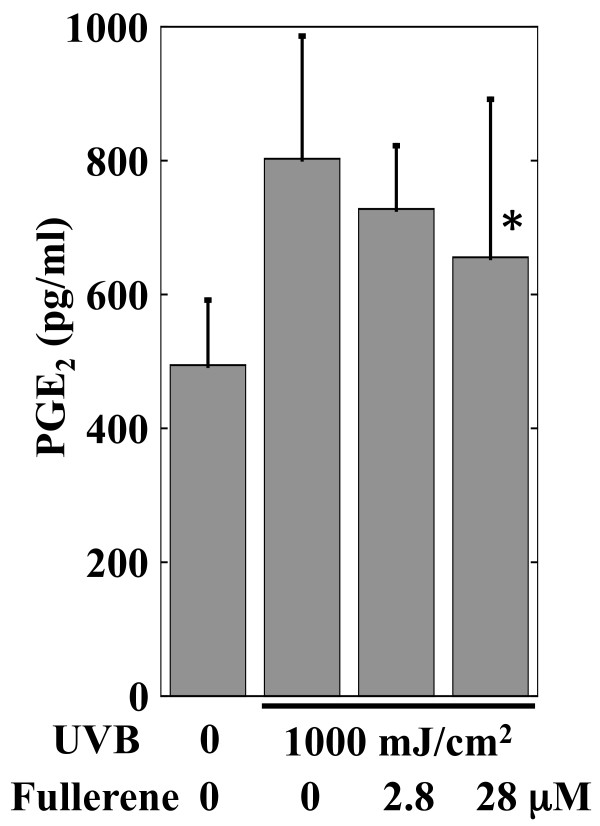
**Effect of fullerene on UVB-induced PGE**_**2 **_**production in the reconstructed human epidermis.** The reconstructed human epidermis was incubated for 1 hour and then 2.8 μM or 28 μM water-soluble polyvinylpyrrolidone (PVP)-wrapped fullerene or a mock solution (1% PVP in PBS) was added. After incubation for 1 hour, the cultures were irradiated with 1000 mJ/cm^2^ UVB. Thirty minutes later, the medium was refreshed and the cultures were continued for 6 hours. Then the conditioned medium was subjected to PGE_2_ ELISA. The graph shows the mean ± standard deviation (n =7 ). *p < 0.05, Mann-Whitney *U*-test.

The mean number of conspicuous facial pores before and after the fullerene lotion treatment is shown in Figure 1a. The fullerene lotion significantly decreased the conspicuous pores by 17.6% (p < 0.05, Wilcoxon signed-rank test) after an 8-week treatment. Because this reduction seemed moderately significant to establish cosmetic efficiency, we administered a self-administered questionnaire, which inquired regarding whether participants felt the effect of the tested lotion on opening and pigmentation of pores, facial skin texture and the general condition of facial skin. Among the 10 subjects enrolled, 7 felt an improvement of texture and general condition of facial skin, and 6 felt that there was improvement of the opening and pigmentation of pores; indicating that the fullerene lotion achieved appreciable cosmetic effect. The representative case is shown in Figure 1b, which demonstrates a reduction in the conspicuous pores on the cheek. Using dermoscopy, the regression of the conspicuous pores (triangles) was observed in the same case (Figure 1c). It has been previously reported that skin pigmentation can be classified into three main patterns, i.e., spotty, globular and elongated types, using a video camera equipped with an UV-emitting unit [3]. Moreover, the spotty type corresponds to a perifollicular pattern where the dark spots are round and evenly distributed, and it is decreased by depigmenting formulations such as azelaic acid and the soy extract, resulting in skin lighting [3]. In addition, we examined the effect of topical APPS on the conspicuous facial pores using another high-resolution digital camera system called the Robo-skin analyser RSA-50^®^ (Inforward Co, Tokyo, Japan) [1]. It was seen that APPS treatment significantly decreased the conspicuous and blackish pores but not the open pores after a 4-week treatment, indicating that APPS can decrease conspicuous pores mainly by whitening them. Collectively, the regression of conspicuous pores can be ascribed to the suppression of perifollicular melanogenesis. Accordingly, to investigate the mechanism of effect of fullerene, its effect was examined on UVB-induced PGE_2_ production in RhE because PGE_2_ reportedly stimulates tyrosinase and dendricity of melanocytes [4,5]. Irradiation with 1000 mJ/cm^2^ increased PGE_2_ production in RhE by 62.3% (p < 0.05, Mann-Whitney *U*-test). However, the addition of 28 μM fullerene significantly suppressed the UVB-induced PGE_2_ production by 18.3% (p < 0.05) (Figure 2). Therefore, we suggest that the suppression of PGE_2_ in the epidermis is one of the potential pathways for the effect of fullerene on melanogenesis, which is associated with the pathogenesis of conspicuous pores. In addition, water-soluble PVP-wrapped fullerene reportedly shows an appreciable absorbance in the UVB spectrum (290–320nm) [6], suggesting that the effect of fullerene on PGE_2_ may be partially through UV absorption.

Here, we utilised the LabCyte EPI-MODEL24 but this model does not contain melanocytes. Therefore, our assays reflected only fullerene effect on epidermal keratinocytes. On the other hand, fullerene has been previously reported to inhibit UVA-induced melanogenesis from cultured human melanocytes [6]. Collectively, keratinocytes and melanocytes both are suggested targets of fullerene. However, to explore the effect of fullerene on PGE_2_ production more precisely, it is of interest to study of animal models or even ex-vivo human skin in the near future.

Because even a dose of 80 mJ/cm^2^ UVB is reportedly sufficient for induction of PGE_2_ production in cultured human keratinocytes [7], the dose of UVB used for irradiation in this study (1000 mJ/cm^2^) was extremely high. Likewise, the mean of minimal erythema dose of UVB for Japanese is 43.7 mJ/cm^2^[8]; therefore, the UVB dose utilised here is remarkably intense. However, previously it was reported that, whereas only 5 mJ/cm^2^ UVB could stimulate co-culture of human keratinocytes and melanocytes, a 100mJ/cm^2^ UVB dose was required to induce melanogenesis in human pigmented reconstructed epidermis. This indicates that the reconstructed epidermis is more resistant to the UVB effect than cultured epidermal cells [9], supporting our observation of resistance or insensitivity of the RhE to UVB.

In conclusion, the fullerene lotion significantly decreased the conspicuous facial pores after an 8-week treatment. Further, the experimental data suggests that the suppression of PGE_2_ production in epidermis is one of potential pathways for this cosmetic effect of fullerene.

## Abbreviations

APPS: Ascorbyl 2-phosphate 6-palmitate; PGE2: Prostaglandin E_2_; RhE: Reconstructed human epidermis; PVP: Polyvinylpyrrolidone.

## Competing interests

The cost of this study was covered by Vitamin C60 BioResearch Corporation. The authors MI and SH belong to this company.

## Authors’ contributions

S Inui and S Itami designed and organized the whole study. S Inui analyzed the data and wrote the paper. AM carried out the clinical study. MI and SH performed the *in vitro* experiments. All authors read and approved the final manuscript.
